# Secondary structures and cell-penetrating abilities of arginine-rich peptide foldamers

**DOI:** 10.1038/s41598-018-38063-8

**Published:** 2019-02-04

**Authors:** Makoto Oba, Yu Nagano, Takuma Kato, Masakazu Tanaka

**Affiliations:** 1 Graduate School Biomedical Sciences, Nagasaki University, 1-14 Bunkyo-machi, Nagasaki, 852-8521 Japan; 2 Osaka University of Pharmaceutical Sciences, 40-20-1 Nasahara, Takatsuki, Osaka 569-1094 Japan

## Abstract

Foldamers, which are folded oligomers with well-defined conformations, have been recently reported to have a good cell-penetrating ability. α,α-Disubstituted α-amino acids are one such promising tool for the design of peptide foldamers. Here, we prepared four types of L-arginine-rich nonapeptides containing L-leucine or α,α-disubstituted α-amino acids, and evaluated their secondary structures and cell-penetrating abilities in order to elucidate a correlation between them. Peptides containing α,α-disubstituted α-amino acids had similar resistance to protease digestion but showed different secondary structures. Intracellular uptake assays revealed that the helicity of peptides was important for their cell-penetrating abilities. These findings suggested that a peptide foldamer with a stable helical structure could be promising for the design of cell-penetrating peptides.

## Introduction

Cell-penetrating peptides (CPPs) are promising tools for the delivery of membrane-impermeable compounds into living cells^1,2^. Arginine (Arg)-rich peptides are one of the most remarkable class of CPPs that can effectively translocate across the cell membrane^3–5^. A cationic amphipathic α-helical peptide is also a representative CPP^6,7^. There have been numerous studies on the development of novel CPPs with more efficient cell-penetrating abilities and lower cytotoxicities than existing CPPs. For this purpose, the use of peptide foldamers is an attractive strategy. The term foldamer has been used to describe an oligomer with a well-defined compact conformation^8–10^. β-Peptide foldamer was used for building a stable cationic amphipathic helix and showed high cell-penetrating ability^11^. A quinoline-based aromatic foldamer was reported to cross the cell membrane despite its anionic nature^12^. It is also worth noting that foldamers are able to deliver not only low-molecular-weight compounds such as fluorescein but also biomacromolecules such as nucleic acids into cells *in vitro*^13–16^. We previously reported CPP foldamers containing α,α-disubstituted α-amino acids (dAAs)^17–21^. α-Methylated and cyclic dAAs are known to stabilize a peptide secondary structure into a helix^22,23^. The introduction of cyclic dAAs into Arg-rich peptides led to a marked conformational change from a random coil to a helical structure and elevated the stability of peptides against protease digestion^24,25^. Increased resistance of Arg-rich peptides to protease digestion in serum resulted in enhanced and prolonged cell-penetrating abilities. However, no definitive evidence that the stable helical structure of peptide foldamers with dAAs, excluding amphipathic helical peptides^18,26,27^, led to their high cell-penetrating abilities has been reported so far.

**Figure 1:**
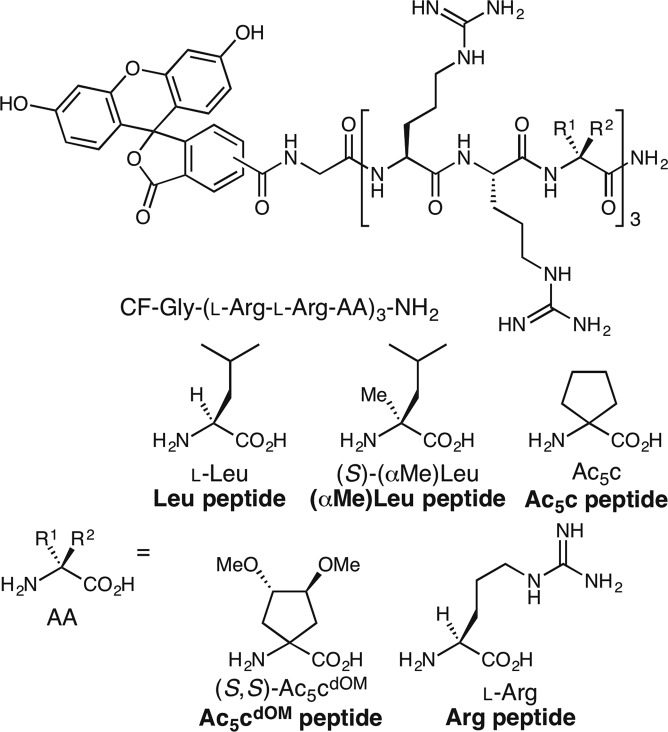
Design of Leu, (αMe)Leu, Ac_5_c, Ac_5_c^dOM^, and Arg peptides used in the present study.

In the present study, we prepared four types of Arg-rich nonapeptides composed of three L-Arg-L-Arg-AA repeating units (AA: amino acid), i.e. peptides containing L-leucine (L-Leu; Leu peptide), (*S*)- α-methylleucine ((*S*)-(αMe)Leu; (αMe)Leu peptide), 1-aminocyclopentanecarboxylic acid (Ac_5_c; Ac_5_c peptide), and (3*S*,4*S*)-1-amino-3,4-dimethoxycyclopentanecarboxylic acid ((*S*,*S*)-Ac_5_c^dOM^; Ac_5_c^dOM^ peptide) (Fig. 1). The positive control was Arg nonapeptide (Arg peptide) as an example of an efficient CPP. In the peptides designed here, three L-Arg in the Arg peptide were replaced with hydrophobic AAs including three types of dAAs. We expected that (αMe)Leu, Ac_5_c, and Ac_5_c^dOM^ peptides (containing α-methylated or cyclic dAAs) would adopt helical structures with a little difference even in aqueous solution. The objective of this study was to investigate the effects of stable helical structures of peptide foldamers after the introduction of dAAs on their cell-penetrating abilities. In order to clarify any influence of dAA incorporation, Leu peptide, which was composed of only natural AAs, was also prepared as a control.

## Results and Discussion

### Preparation of peptides

**Figure 2:**
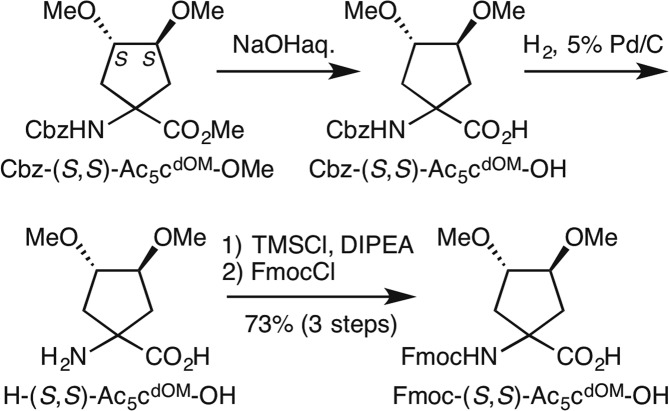
Synthesis of Fmoc-(*S*,*S*)-Ac_5_c^dOM^-OH.

9-Fluorenylmethoxycarbonyl (Fmoc)-protected dAA, Fmoc-(*S*,*S*)-Ac_5_c^dOM^-OH, was synthesized according to Fig. 2 from Cbz-(*S*,*S*)-Ac_5_c^dOM^-OMe, which was prepared from dimethyl L-(+)-tartrate, as reported previously^28,29^. The hydrolysis of Cbz-(*S*,*S*)-Ac_5_c^dOM^-OMe under alkaline conditions followed by deprotection of the Cbz-protecting group by hydrogenolysis using H_2_ and 5% Pd/C gave crude dAA H-(*S*,*S*)-Ac_5_c^dOM^-OH. The primary amine of H-(*S*,*S*)-Ac_5_c^dOM^-OH was protected by Fmoc in order to afford Fmoc-protected dAA, Fmoc-(*S*,*S*)-Ac_5_c^dOM^-OH for solid-phase synthesis, with a yield of 73% over 3 steps (Fig. 2).

Peptides were prepared using the Fmoc solid-phase method with (1-cyano-2-ethoxy-2-oxoethylidenaminooxy)dimethylamino-morpholino-carbenium hexafluorophosphate (COMU), *O*-(7-azabenzotriazol-1-yl)-1,1,3,3-tetramethyluronium hexafluorophosphate (HATU)/1-hydroxy-7-azabenzotriazole (HOAt), or 1-[bis(dimethylamino)methylene]-1*H*-benzotriazolium 3-oxide hexafluorophosphate (HBTU)/1-hydroxybenzotriazole (HOBt) as the coupling reagents. Fmoc-dAA and subsequent Fmoc-AA were introduced into the peptides with double coupling, and capping of the unreacted N-terminal amine was performed using acetic anhydride after each coupling reaction. Carboxyfluorescein (CF) was introduced as a fluorescent label via a glycine (Gly) linker on resin in order to monitor the peptides being internalizing into cells. Peptides that cleaved from the resin were purified with reverse-phase HPLC (RP-HPLC). The homogeneities and purities of the peptides were verified by analytical RP-HPLC and matrix-assisted laser desorption-ionization time-of-flight mass spectrometry (MALDI-TOF-MS) (Fig. S2). Arg peptide was prepared as reported previously^24,30^. Peptides synthesized here readily dissolved in aqueous solution.

### Secondary structures of peptides

**Figure 3:**
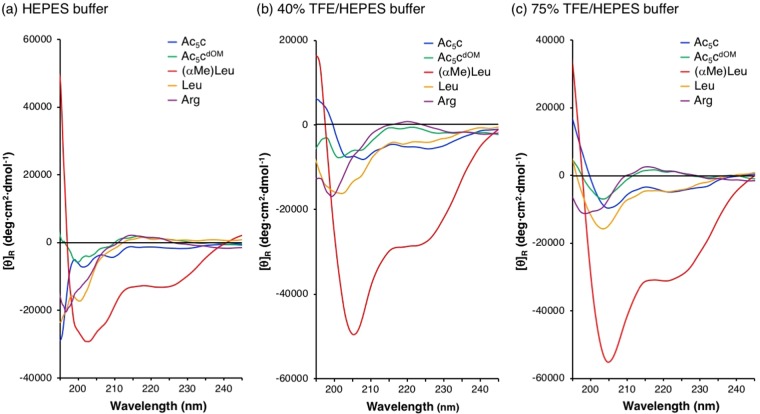
CD spectra of peptides in (**a**) HEPES buffer, (**b**) 40% TFE/HEPES buffer, and (**c**) 75% TFE/HEPES buffer. Peptide concentration: 24 µM.

The conformations of peptides were analyzed by circular dichroism (CD) in pH 7.3 4-(2-hydroxyethyl)-1-piperazineethanesulfonic acid (HEPES) buffer (Fig. 3a), 40% 2,2,2-trifluoroethanol (TFE)/pH 7.3 HEPES buffer (Fig. 3b), and 75% TFE/pH 7.3 HEPES buffer (Fig. 3c). Negative maxima at 205–209 nm (*π* → π*) and 222–225 nm (*n* → π*) were diagnostic of a right-handed (*P*) helical structure^31–33^. The ratio of *R* (*θ*_*π* → π*_/*θ*_*n* → π*_) has been used as a parameter to distinguish an α-helical structure from a 3_10_-helical structure (i.e., *R* ≈ 1: α-helix; *R* < 0.4: 3_10_-helix) (Table S1)^34,35^. Helical content (helicity) of peptides was estimated from the intensity of *θ*_*n* → π*_ values^36^. A negative maximum at 195–200 nm (*π* → π*) and a weak positive maximum at 217 nm (*n* → π*) were diagnostic of a random coil structure. In HEPES buffer (Fig. 3a), the spectra of Ac_5_c^dOM^, Leu, and Arg peptides were similar to those of a typical random coil structure, whereas (αMe)Leu peptide showed negative maxima at 203 nm and 223 nm, suggesting the inclusion of a right-handed (*P*) helical structure. α-Methylated dAA ((αMe)Leu)) contributed to formation of a more stable helical structure of Arg-rich peptides than cyclic dAAs (Ac_5_c and (*S*,*S*)-Ac_5_c^dOM^). By adding TFE to HEPES buffer, (αMe)Leu peptide formed a complete right-handed *(P*) helical structure in a mixture of α- and 3_10_-helices (*R* = 0.57 and 0.56 in Figs 3b and 2c, respectively). The dominant structures of Ac_5_c and Leu peptides changed by increased the rate of TFE to eventually be α-/3_10_-helices (*R* = 0.52) and 3_10_-helix (*R* = 0.30), respectively (Fig. 3c). (αMe)Leu, Ac_5_c, and Leu peptides formed right-handed (*P*) helical structures in 75% TFE/HEPES buffer, but the intensity of their CD spectra varied markedly, indicating that the stability of right-handed (*P*) helical structures was highest for (αMe)Leu peptide. Contrary to expectations, the Ac_5_c^dOM^ peptide showed a negative maximum at 203 nm and a positive maximum at 217 nm (Fig. 3c); thus, its dominant structure appeared to be a random structure similar to Arg peptide even after adding TFE. Cbz-[L-Leu-L-Leu-(*S*,*S*)-Ac_5_c^dOM^]_3_-OMe was reported to form a right-handed (*P*) helical structure in 50% TFE/H_2_O^37^. L-Leu might contribute to stabilization of the peptide helical secondary structure as well as (*S*,*S*)-Ac_5_c^dOM^. Destabilization of the peptide helical secondary structure by L-Arg might be stronger than stabilization of it by (*S*,*S*)-Ac5c^dOM^. Taken together with the results of CD spectra (Fig. 3), in which helicity of peptides was estimated from the intensity of *θ*_*n* → π*_ values^36^, stability in the helicity of the peptides in the current study was as follows:

$$({\rm{\alpha }}\mathrm{Me}){\rm{Leu}}\,{\rm{peptide}} > {{\rm{Ac}}}_{5}{\rm{c}}\,{\rm{peptide}}\approx {\rm{Leu}}\,{\rm{peptide}} > {{\rm{Ac}}}_{5}{{\rm{c}}}^{{\rm{dOM}}}{\rm{peptide}}\approx {\rm{Arg}}\,{\rm{peptide}}.$$

### Peptide stability against protease digestion

**Figure 4:**
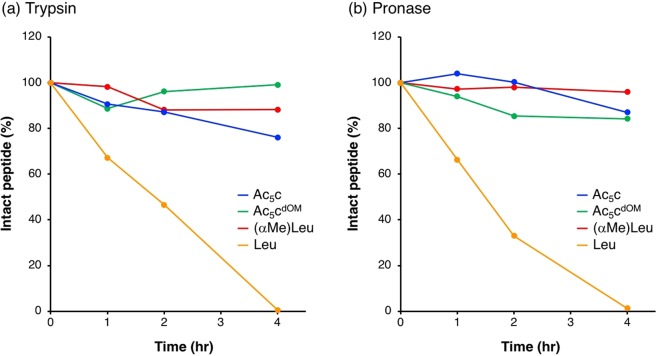
Enzymatic degradation of peptides using (**a**) trypsin and (**b**) pronase.

Next, we examined the stability of peptides against protease digestion using trypsin (Fig. 4a) and pronase (Fig. 4b). One of the advantages of peptide foldamers containing dAAs is their high resistance to degradation by proteases^38–40^. Trypsin is a serine protease that cleaves amide bonds at the C terminus of cationic amino acids such as Arg and lysine. Pronase is a non-specific protease mixture produced in the culture supernatant of *Streptomyces griseus*. Ac_5_c, Ac_5_c^dOM^, (αMe)Leu, and Leu peptides were incubated with trypsin or pronase for 1, 2, and 4 h, and then rate of intact peptide was evaluated by RP-HPLC using α-cyano-4-hydroxycinnamic acid (α-CHCA) as an internal standard. Leu peptide was promptly degraded by both trypsin and pronase, and reached 0% intact peptide after 4 h incubation in a similar manner to Arg peptide reported previously^24,25^. On the other hand, approximately 80% of Ac_5_c, Ac_5_c^dOM^, and (αMe)Leu peptides remained intact even after 4 h incubation. Introduction of dAAs into peptides markedly elevated resistance to protease digestion, whether or not peptides formed stable helical structures. In other words, peptides composed of only natural α-amino acids were easily degraded by proteases independently of their peptide secondary structures.

### Cellular uptake of peptides

**Figure 5:**
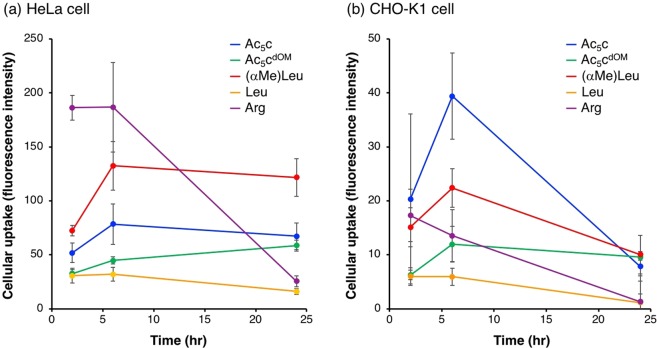
Cellular uptake of peptides into (**a**) HeLa cells and (**b**) CHO-K1 cells.

Cellular uptake of peptides was assessed against HeLa (Fig. 5a) and CHO-K1 cells (Fig. 5b) at a concentration of 1 µM and various incubation times (2, 6, and 24 h). In HeLa cell, the cellular uptake of Arg peptide was the best under conditions of shorter incubation times (2 and 6 h) but markedly decreased after 24 h incubation. A small decrease was also observed in Leu peptide. On the other hand, Ac_5_c and (αMe)Leu peptides showed increased cellular uptake from 2 to 6 h incubations and this was maintained after 24 h incubation. Furthermore, the cellular uptake of Ac_5_c^dOM^ peptide increased up to 24 h. Similar tendencies that peptides containing dAAs (Ac_5_c, Ac_5_c^dOM^, and (αMe)Leu peptides) showed prolonged cell-penetrating ability compared with peptides composed of only natural α-amino acids (Arg and Leu peptides) were also observed in CHO-K1 cells (Fig. 5b). These results might be attributed to different stabilities against protease digestion (Fig. 4) in medium containing fetal bovine serum (FBS). However, although Ac_5_c, Ac_5_c^dOM^, and (αMe)Leu peptides showed almost the same stabilities against trypsin and pronase digestion (Fig. 4), Ac_5_c^dOM^ peptide showed the most prolonged cell-penetrating ability. Not only stabilities against protease digestion but also other factors might contribute to prolongation of the cell-penetrating ability of peptides. Surprisingly, cellular uptake of Ac_5_c, Ac_5_c^dOM^, and (αMe)Leu peptides into both cells was higher than that of Arg peptide after 24 h incubation, and that of Ac_5_c and (αMe)Leu peptides into CHO-K1 cells was higher than that of Arg peptide even after a shorter incubation (6 h). The number of Arg residues in Arg-rich peptides is very important for their cell-penetrating ability, and six Arg are not enough^41^. We also reported that (L-Arg-L-Arg-Aib)_3_ nonapeptide (Aib: aminoisobutyric acid, which is the simplest dAA with two methyl groups at the α-position) showed much lower cell-penetrating ability than Arg nonapeptide against HeLa cells after 24 h incubation^19^. Substitution of Arg in Arg peptide with Ac_5_c, Ac_5_c^dOM^, or (αMe)Leu positively affected its cell-penetrating ability compared with Aib. In comparison, among the peptides containing dAAs, (αMe)Leu peptide was the best for HeLa cell, Ac_5_c peptide was the best for CHO-K1 cell, and Ac_5_c^dOM^ peptide was the worst for both cell types (Fig. 5). Although the best peptides were different depending on cell type, the worst peptide was same for both cell types. These results implied that a certain level of helicity contributed to high cell-penetrating ability. Ac_5_c^dOM^ peptide adopting a random structure showed higher cellular uptake than Leu peptide adopting a helical structure at longer incubation times, which might not be due to the secondary structure but resistance to protease degradation. Taken together, a stable helical structure by introducing dAAs into Arg-rich peptide enhanced their cell-penetrating abilities. Peptides with high cell-penetrating ability sometimes possessed high cytotoxicity, but HeLa and CHO-K1 cells treated with each peptide for 2 h did not exhibit any cytotoxicity at concentrations up to 2 µM (Fig. S3). Furthermore, the cultured cells under experimental conditions in Fig. 5 showed no cellular morphological change and no decrease in cell number by microscopic observations.

### Confocal laser scanning microscopic (CLSM) observation of peptides

**Figure 6:**
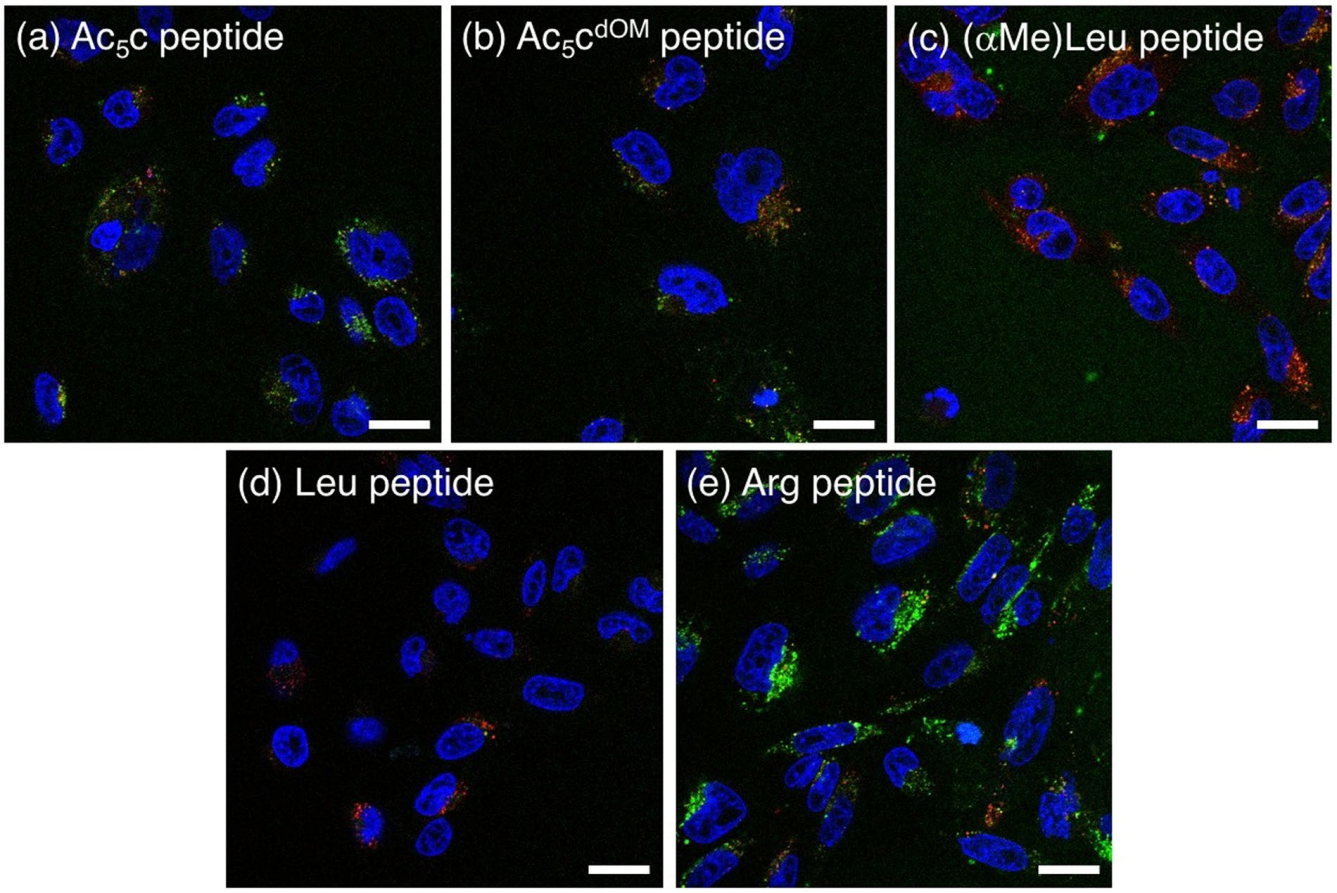
CLSM images of CHO-K1 cells treated with (**a**) Ac_5_c peptide, (**b**) Ac_5_c^dOM^ peptide, (**c**) (αMe)Leu peptide, (**d**) Leu peptide, and (**e**) Arg peptide (green) with late endosomes/lysosomes (red) and nuclei (blue) stained using LysoTracker Red and Hoechst 33342, respectively. Scale bars represent 20 µm.

In order to elucidate the cellular uptake mechanism of peptides, CLSM observation of each peptide (green) was conducted against CHO-K1 cells at a concentration of 1 µM and 2-h incubation with staining of late endosomes/lysosomes (red) and nuclei (blue) (Fig. 6). All peptides were observed not as diffuse signals in the cytoplasm but as green or yellow spots. Peptides containing dAAs appeared to be internalized into cells not via direct penetration of the cell membrane but via endocytic routes. The colocalization of the peptides with late endosomes/lysosomes was quantified and is shown in Fig. S5. Significant differences were observed between Arg peptide and Ac_5_c/(αMe)Leu peptides (*P* < 0.001 for Ac_5_c peptide; *P* < 0.0001 for (αMe)Leu peptide), and therefore, final destinations of Arg peptide and Ac_5_c/(αMe)Leu peptides in CHO-K1 cells appeared to be different. These differences might lead to stronger green signals for Arg peptide than Ac_5_c/(αMe)Leu peptides in Fig. 6.

### Inhibition of cellular uptake of peptides by specific endocytosis inhibitors

To obtain further information on the cellular uptake mechanisms of peptides, inhibitory experiments were carried out using specific endocytosis inhibitors (amilorilde: macropinocytosis; sucrose: clathrin-mediated endocytosis; nystatin: caveolae-mediated endocytosis) against CHO-K1 cells (Fig. S5). The uptake of Ac_5_c and (αMe)Leu peptides was significantly decreased by the treatment with three (amiloride; sucrose; nystatin) and two inhibitors (sucrose; nystatin), respectively. (αMe)Leu peptide appeared to internalize into CHO-K1 cells via all three routes, while Ac_5_c peptide appeared to internalize mainly via clathrin-mediated endocytosis and caveolae-mediated endocytosis. Furthermore, three inhibitors could not completely inhibit the uptake of each peptide, suggesting the existence of other uptake mechanisms such as direct penetration and/or different type of endocytosis.

In summary, we investigated the cell-penetrating abilities of Arg-rich peptides containing dAAs from the viewpoint of peptide secondary structures. Introduction of α-methylated or cyclic dAAs into Arg-rich peptides led to various peptide secondary structures from helical to random structures regardless of similar stability against protease digestion. The helicity of the peptides correlated roughly with their cell-penetrating abilities. These findings will provide a strategy for designing better CPPs.

## Methods

### General

Optical rotation [α]^rt^_D_ was measured using a Jasco DIP-370 polarimeter (JASCO, Tokyo, Japan) using a 0.5 dm cell. Infrared (IR) spectra were recorded using a Shimadzu IR Affinity-1 spectrometer (Shimadzu Corporation, Kyoto, Japan) for conventional measurement (KBr). CD spectra were recorded using a Jasco J-720W spectropolarimeter (JASCO) with a 1.0 mm path length cell. ^1^H NMR (400 MHz) and ^13^C NMR (100 MHz) spectra were determined using a JEOL AL 400 spectrometer (JEOL Ltd, Tokyo, Japan). Fast atom bombardment (FAB)-MS was measured using a JEOL JMS-700N (JEOL). MALDI-TOF-MS spectra were recorded using a Bruker Ultrax spectrometer (Bruker Daltonics, Fermont, CA). Piperidine was purchased from Tokyo Chemical Industry Co., Ltd. (Tokyo, Japan). Fmoc-L-Arg(Pbf)-OH, Fmoc-Gly-OH, Fmoc-L-Leu-OH, and CLEAR-Amide resin were purchased from the Peptide Institute., Inc. (Osaka, Japan). Fmoc-(*S*)-(αMe)Leu-OH was obtained from Nagase & Co., Ltd. (Kyoto, Japan). Fmoc-Ac_5_c-OH was purchased from Watanabe Chemical Industries, Ltd. (Hiroshima, Japan). HATU, HBTU, HOAt, and HOBt were from AAPPTec (Louisville, KY). COMU was from Novabiochem (Tokyo, Japan). CF and Dulbecco’s modified Eagle’s medium (DMEM) were obtained from Sigma-Aldrich Co. (St. Louis, MO). diisopropylethylamine (DIPEA) and heparin were purchased from Wako Pure Chemicals Co., Inc. (Osaka, Japan). Diethyl ether was from Kanto Chemical Co., Inc. (Tokyo, Japan). Trifluoroacetic acid (TFA) was obtained from Nacalai Tesque, Co., Inc. (Kyoto, Japan). Hoechst 33342 and a Cell counting kit-8 were purchased from Dojindo Laboratories (Kumamoto, Japan). LysoTracker Red was from Molecular Probes (Eugene, OR).

### Synthesis of Fmoc-(*S*,*S*)-Ac_5_c^dOM^-OH

A solution of 0.1 M aqueous NaOH (35 mL, 3.5 mmol) was added to a stirred solution of Cbz-(*S*,*S*)-Ac_5_c^dOM^-OMe (808 mg, 2.4 mmol) in MeOH (30 mL) at 0 °C. After stirring at room temperature for 12 h, the solution was neutralized with 1 M aqueous HCl, evaporated to remove MeOH, extracted with EtOAc, dried over Na_2_SO_4_, and evaporated in vacuo to leave a crude carboxylic acid Cbz-(*S*,*S*)-Ac_5_c^dOM^-OH (776 mg, quantitative). A mixture of the crude carboxylic acid Cbz-(*S*,*S*)-Ac_5_c^dOM^-OH (776 mg, 2.4 mmol) and 5% Pd/C (520 mg) in MeOH (80 mL) was vigorously stirred in an H_2_ atmosphere at room temperature for 24 h. The Pd/C catalyst was filtered off, and the filtrate was evaporated to give crude amino acid H-(*S*,*S*)-Ac_5_c^dOM^-OH (429 mg, quantitative). A mixture of the crude amino acid H-(*S*,*S*)-Ac_5_c^dOM^-OH (429 mg, 2.3 mmol) and DIPEA (1.4 mL, 7.9 mmol) in CH_2_Cl_2_ (30 mL) was stirred at room temperature for 30 min, and then, trimethylsilyl chloride (0.58 mL, 4.5 mmol) was added. The solution was stirred at room temperature for 30 min and refluxed for 4 h. After cooling to −20 °C, Fmoc-Cl (0.58 mL, 4.5 mmol) was added to a stirred solution and the solution was stirred at room temperature for 18 h. The solution was acidified with 1% aqueous HCl, extracted with CHCl_3_, and dried over MgSO_4_. Removal of the solvent afforded a residue, which was purified by column chromatography on silica gel (10% MeOH in CHCl_3_) to give Fmoc-(*S*,*S*)-Ac_5_c^dOM^-OH (684 mg, 3 steps 73%) as colorless crystals. M.p. 65–67 °C; [α]^25^_D_ = + 28.3 (*c* 1.1, CHCl_3_); IR (KBr) 3433 (br), 2936, 1724, 1502, 1244 cm^–1^; ^1^H NMR (400 MHz, CDCl_3_) δ 7.88 (br s, 1H), 7.72 (d, *J* = 7.7 Hz, 2H), 7.56 (d, *J* = 6.8 Hz, 2H), 7.37 (t, *J* = 7.3 Hz, 2H), 7.26 (t, *J* = 7.3 Hz, 2H), 4.39 (m, 2H), 4.20 (t, *J* = 6.8 Hz, 1H), 3.84 (m, 2H), 3.33 (s, 6H), 2.50 (m, 2H), 2.32 (m, 1H), 2.08 (d, *J* = 14 Hz, 1H); ^13^C NMR (100 MHz, CDCl_3_) δ 176.9, 155.5, 143.8, 143.6, 141.2 (2C), 127.6 (2C), 125.1 (2C), 119.9 (2C), 85.2, 85.1, 66.8, 63.6, 57.2, 57.1, 47.1, 40.3, 40.1; HRMS (FAB(+)) calcd for C_23_H_25_NO_6_ [M + H]^+^ 412.1760, found 412.1763.

### Synthesis and characterization of peptides

The peptides were synthesized on solid support by Fmoc solid-phase methods using standard commercially available Rink amide resin and Fmoc-amino acids. The following describes a representative coupling and deprotection cycle at 50 μ mol scales. First, 100 mg of CLEAR-Amide resin (loading: 0.50 mmol/g) was soaked overnight in *N*,*N*-dimethylformamide (DMF). DMF had been removed, and 20% piperidine in DMF was added to the resin for deprotection. After removing and washing out piperidine, Fmoc-amino acid or 5(6)-CF (3 equiv), coupling reagents (COMU, HATU/HOAt, or HBTU/HOBt) (3 equiv), and DIPEA (6 equiv) dissolved in DMF (2.0 mL) were added for the coupling reaction. Fmoc-dAAs and Fmoc-L-Arg(Pbf)-OH next to dAA were introduced into the peptides with double coupling. Capping of the unreacted N-terminal aminewas carried out using acetic anhydride. The resin was suspended in cleavage cocktail (TFA/TIPS/H_2_O: 95/2.5/2.5). The TFA solution was evaporated to a small volume and added to cold diethyl ether to precipitate the peptides. The dried crude peptides were dissolved in CH_3_CN and/or H_2_O, and then purified by RP-HPLC using a COSMOSIL Packed Column 5C_18_-AR-II (20 ID × 250 mm). Freeze-drying afforded yellow crystals, which were characterized by analytical RP-HPLC (COSMOSIL Packed Column 5C_18_-AR-II 4.6 ID × 250 mm) and MALDI-TOF-MS. RP-HPLC was performed using a JASCO-PU-2086 Plus with UV-2075 Plus as a detector. Solvent A: 0.1% TFA in H_2_O; solvent B: 0.1% TFA in CH_3_CN. The purification procedure required gradient conditions (from 95% to 5% solvent B in solvent A over 30 min) with a flow rate of 8 mL/min and detection at 220 nm. The purity of the final compounds was confirmed using RP-HPLC conditions (from 95% to 5% solvent B in solvent A over 30 min) with a flow rate of 1.0 mL/min.

### Resistance to proteases

One-hundred microliters of peptide solution (10 µM) in 2.5 × 10^−6^ w/v% trypsin or pronase/phosphate buffer saline (PBS) was incubated at 37 °C for 0, 1, 2, and 4 h. After each incubation time, 250 µL of 1% TFA/PBS solution was added to inactivate the proteases, and 50 µL of 1/2 diluted saturated α-CHCA solution was added as an internal standard, followed by HPLC analysis, which was performed using the above-mentioned conditions.

### Cellular uptake

HeLa or CHO-K1 cells were seeded on 24-well culture plates (100,000 cells/well) and incubated in 400 µL of DMEM containing 10% FBS. The medium was then replaced with fresh medium, and a peptide solution was added to each well at a concentration of 1 µM. After each incubation time, the medium was removed and cells were washed with ice-cold PBS with heparin (20 units/mL) and trypsinized. After the addition of medium containing 10% FBS, fluorescence intensity was detected and acquired using an On-chip Flow (On-chip Biotechnologies Co., Ltd, Tokyo, Japan). The results are presented as a mean and standard deviation obtained from 3 samples.

### Cell viability

HeLa or CHO-K1 cells were seeded on 96-well culture plates (12,000 cells/well) and incubated in 100 µL of DMEM containing 10% FBS. The medium was then replaced with fresh medium, and a peptide solution was added to each well at various concentrations (0.5, 1, and 2 µM). After a 2-h incubation, a Cell counting kit-8 was used in accordance with the manufacture’s protocol. Cell viability was evaluated on the basis of the absorbance of formazan from each well, and 100% cell viability was calculated from wells without peptides. The results are presented as a mean and standard deviation obtained from 4 samples.

### CLSM observation

HeLa or CHO-K1 cells were seeded on 8-well chambered cover glasses (20,000 cells/well) and incubated overnight in 200 µL of DMEM containing 10% FBS. The medium was replaced with fresh medium, and the peptides were applied to each well at a concentration of 1 µM. After a 2-h incubation, the medium was removed and the cells were washed 3 times with ice-cold PBS supplemented with heparin (20 units/mL). The intracellular distribution of CF-labeled peptides (green) was observed by CLSM after staining late endosomes/lysosomes (red) with LysoTracker Red and nuclei (blue) with Hoechst 33342. CLSM observations were conducted using an LSM 710 (Carl Zeiss, Oberlochen, Germany) with a Plan-Apochromat 63×/1.4 objective (Carl Zeiss) at an excitation wavelength of 405 nm (UV laser) for Hoechst 33342, 488 nm (Ar laser) for peptides, and 543 nm (He–Ne laser) for LysoTracker Red, respectively. The rate of colocalization ratio was quantified. The colocalization ratio was quantified as follows:

$${\rm{Colocalization}}\,{\rm{ratio}}( \% )={\rm{peptide}}\,{{\rm{pixels}}}_{{\rm{colocalization}}}/\mathrm{peptide}\,{{\rm{pixels}}}_{{\rm{total}}}\times 100$$

where peptide pixels_colocalization_ represents the number of peptide pixels colocalizing with LysoTracker Red in the cell, and peptide pixels_total_ represents the number of all the peptide pixels in the cell. The results are presented as a mean and standard deviation obtained from 16 cells.

### Inhibition of endocytosis

CHO-K1 cells were seeded on 24-well culture plates (100,000 cells/well) and incubated in 400 µL of DMEM containing 10% FBS. The medium was then replaced with fresh medium containing 10% FBS in the absence (untreated) or presence of amiloride (5 mM), sucrose (0.4 M), or nystatin (25 µg/mL), cells were pre-incubated at 37 °C for 30 min. A peptide solution was added to each well at a concentration of 1 µM. The medium was removed after 1-h incubation and subsequent operations were conducted in the same manner as cellular uptake experiments.

## Supplementary information

Supplementary information
