# Supplementary material for: Secondary structures and cell-penetrating abilities of arginine-rich peptide foldamers
Source: Sci Rep. 2019 Feb 4;9:1349. doi: 10.1038/s41598-018-38063-8 (PMC6362038; doi:10.1038/s41598-018-38063-8)
Supplement: Supplementary file 1 — Supplementary information [file 41598_2018_38063_MOESM1_ESM.pdf]

Secondary structures and cell-penetrating abilities of arginine-rich peptide foldamers

Makoto Oba[1]*, Yu Nagano[1], Takuma Kato[1,2], Masakazu Tanaka[1]

[1]Graduate School Biomedical Sciences, Nagasaki University, 1-14 Bunkyo-machi, Nagasaki 852-8521, Japan

[2]Osaka University of Pharmaceutical Sciences, 40-20-1 Nasahara, Takatsuki, Osaka 569-1094, Japan

(a)

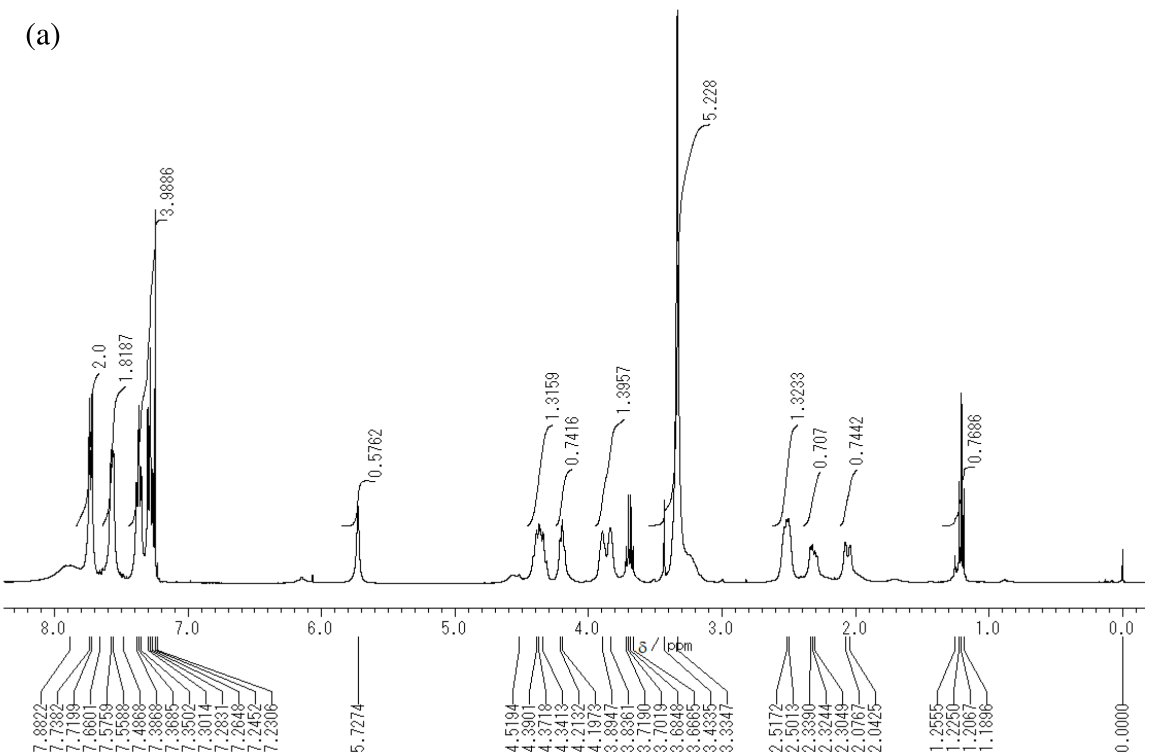

(b)

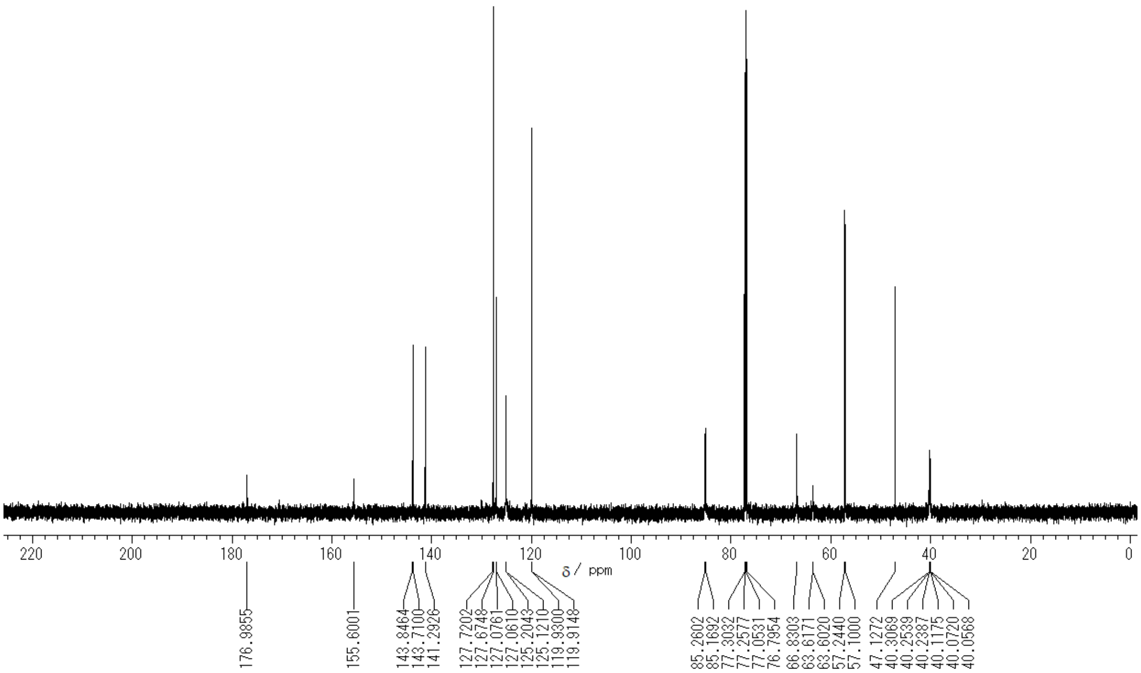

Figure S1. ^{1}H NMR spectrum (a) and ^{13}C NMR spectrum (b) of Fmoc-(*S*,*S*)-Ac_5c^{dOM}-OH.

(a) Leu peptide

MALDI-TOF-MS: *m/z* calcd for $C_{77}H_{122}N_{29}O_{16}$ $[M+H]^+$ 1708.96; found 1709.23

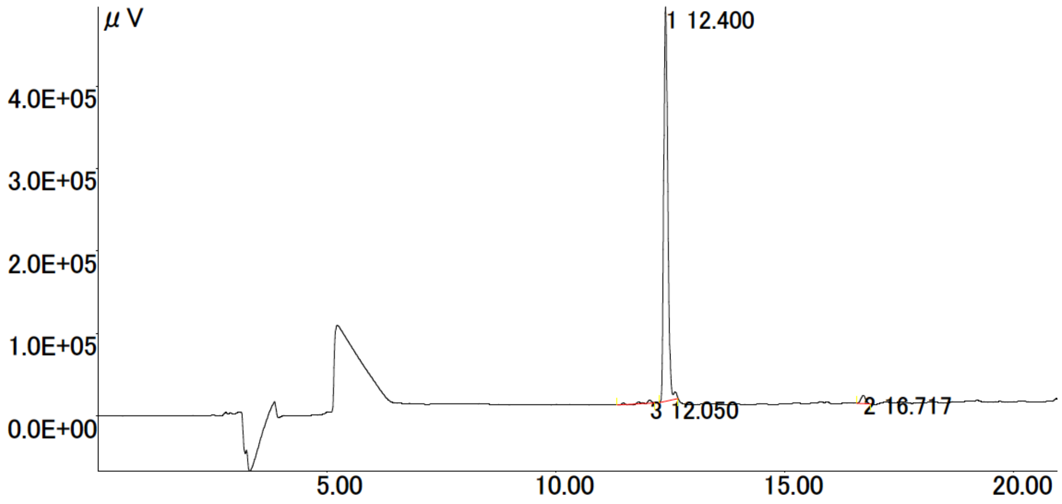

(b) (αMe)Lue peptide

MALDI-TOF-MS: *m/z* calcd for $C_{80}H_{128}N_{29}O_{16}$ $[M+H]^+$ 1751.01; found 1751.98

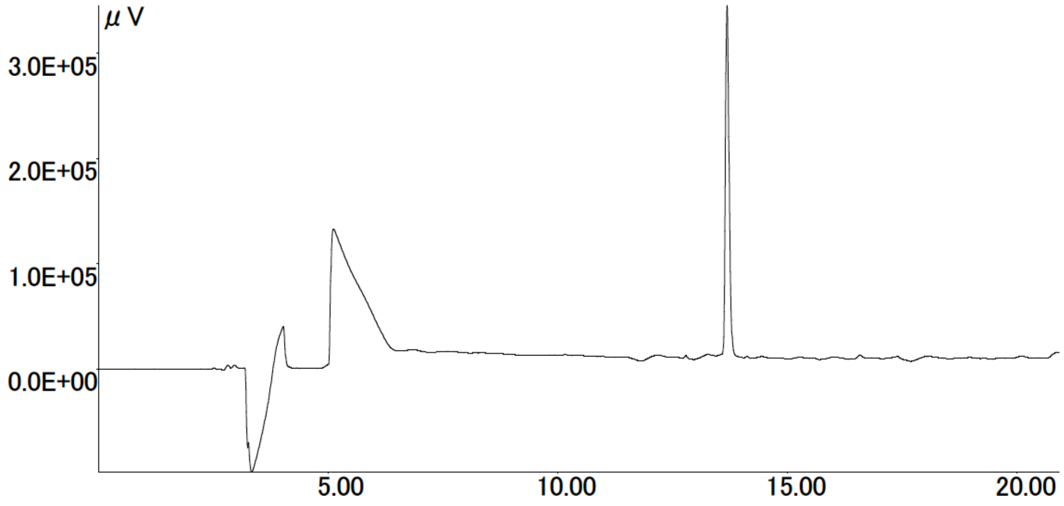

(c) Ac$_5$c peptide

MALDI-TOF-MS: *m/z* calcd for $C_{77}H_{116}N_{29}O_{16}$ $[M+H]^+$ 1702.92; found 1703.02

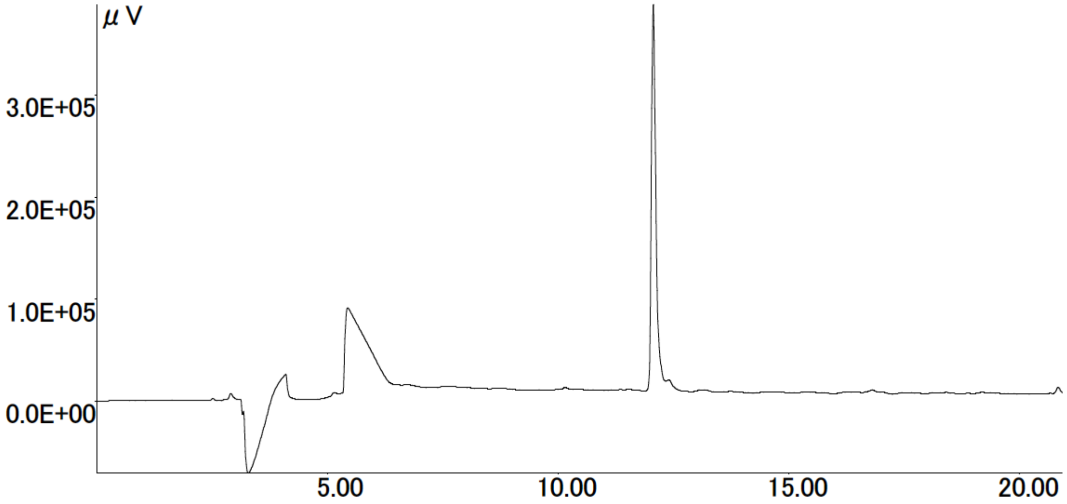

(d) Ac$_5$c^{dOM} peptide

MALDI-TOF-MS: *m/z* calcd for $C_{83}H_{128}N_{29}O_{22}$ $[M+H]^+$ 1882.98; found 1883.42

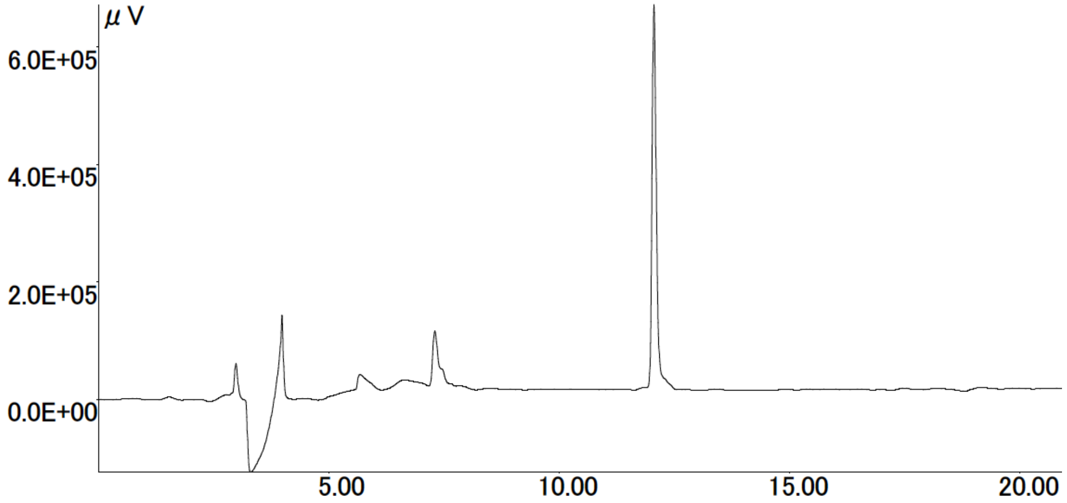

Figure S2. HPLC charts and results of MALDI-TOF-MS of Leu peptide (a), (αMe)Lue peptide (b), Ac$_5$c peptide (c), and Ac$_5$c^{dOM} peptide (d).

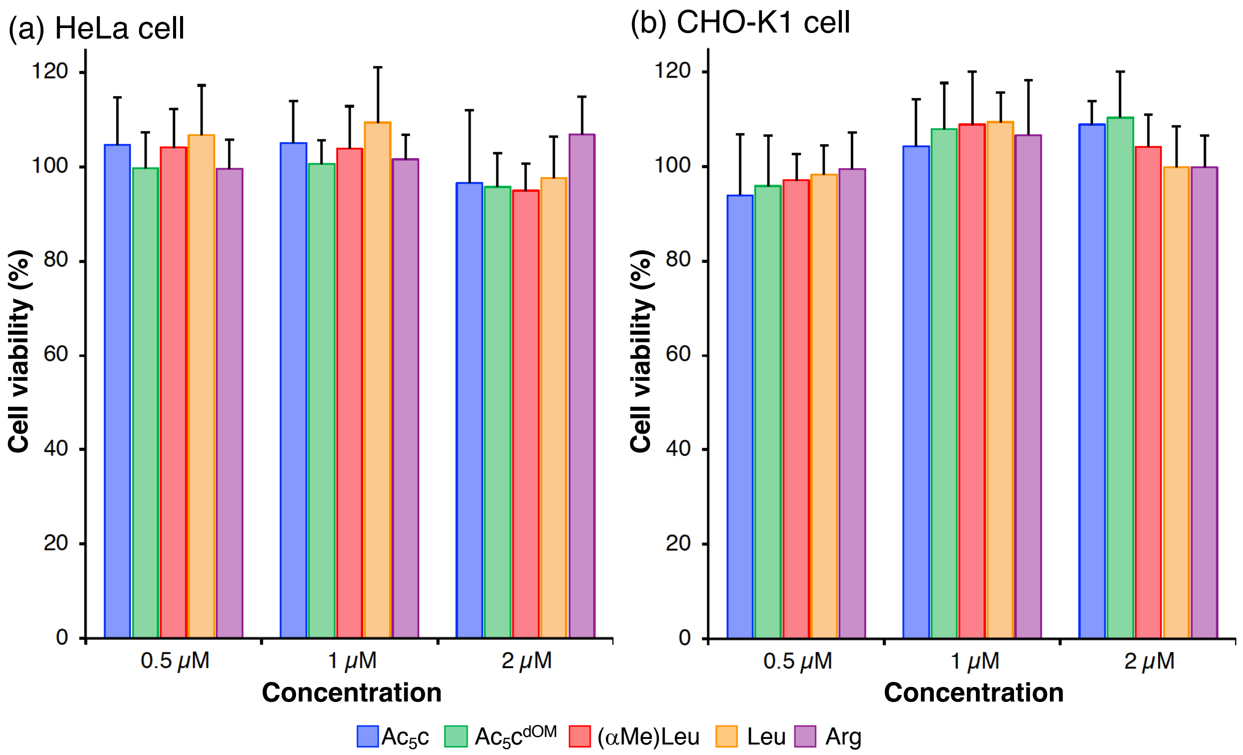

Figure S3. Cell viability of HeLa cell (a) and CHO-K1 cell (b) treated with peptides at concentration of 0.5, 1, and 2 μM for 2 h. Error bars represent the standard deviation, n = 4.

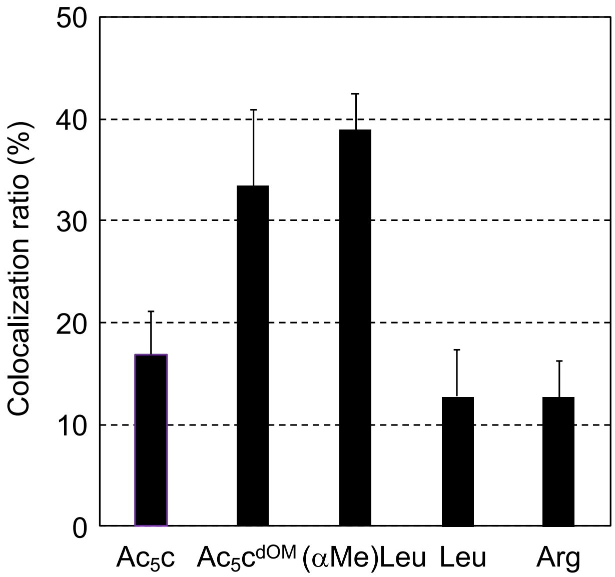

Figure S4. Quantification of the colocalization of peptides with LysoTracker Red. Error bars represent the standard deviation, $n = 16$.

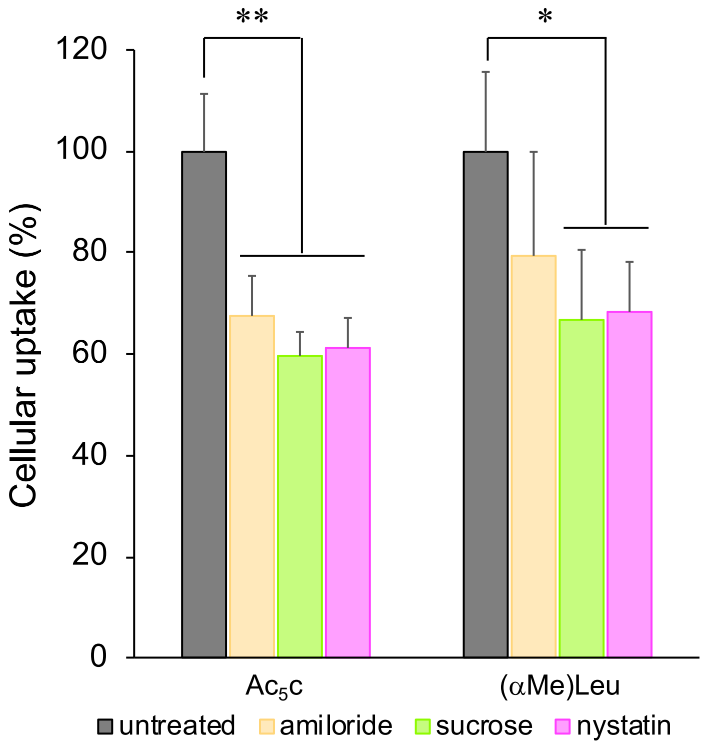

Figure S5. Effects of inhibitors on internalization of peptides against CHO-K1 cells. Error bars represent the standard deviation, $n = 3$. *$P < 0.05$, **$P < 0.01$.

Table S1. *R* values ($\theta_{\pi\rightarrow\pi^*}/\theta_{n\rightarrow\pi^*}$) of synthetic peptides.

Peptide	*R* value ($\theta_{\pi\rightarrow\pi^*}/\theta_{n\rightarrow\pi^*}$)		
	HEPES	40% TFE/HEPES	75% TFE/HEPES
Ac_5c peptide	–[a]	0.61	0.52
Ac_5c^{dOM} peptide	–[a]	–[a]	–[a]
(αMe)Leu peptide	0.45	0.57	0.56
Leu peptide	–[a]	0.26	0.30
Arg peptide	–[a]	–[a]	–[a]

[a]No significant maxima at $\theta_{\pi\rightarrow\pi^*}$ and $\theta_{n\rightarrow\pi^*}$ were observed.
